# Mitochondrial Disease as a Cause of Neonatal Hemophagocytic Lymphohistiocytosis

**DOI:** 10.1155/2016/3932646

**Published:** 2016-09-26

**Authors:** Kazumasa Fuwa, Mitsuru Kubota, Masami Kanno, Hiroshi Miyabayashi, Ken Kawabata, Keiichi Kanno, Masaki Shimizu

**Affiliations:** ^1^Division of Neonatology, Saitama Children's Medical Center, 2100 Magome, Iwatsuki-ku, Saitama 339-8551, Japan; ^2^Department of General Pediatrics & Interdisciplinary Medicine, National Center for Child Health and Development, 2-10-1 Okura, Setagaya-ku, Tokyo 157-8535, Japan

## Abstract

Diagnosis of mitochondrial respiratory chain disorder (MRCD) is often difficult. Its pathogenesis is still unclear. We diagnosed MRCD by measuring the activity of the mitochondrial respiratory chain enzyme, and the patient also had hemophagocytic lymphohistiocytosis (HLH). A preterm female infant was born at 34 weeks of gestation. On day 6, HLH was revealed by bone marrow aspiration. She died on day 10 due to uncontrollable HLH. An autopsy was performed, and we measured the activity of the mitochondrial respiratory chain enzyme in the liver, muscle, and heart. The activity of complex I was decreased in all tissues. As we could not prove another origin of the HLH, she was diagnosed as having HLH caused by MRCD. It is useful to measure the activity of the mitochondrial respiratory chain enzyme for diagnosing MRCD. MRCD, which has a severe clinical course, may be related to HLH.

## 1. Introduction

Mitochondrial respiratory chain disorders (MRCDs) are dysfunction of the oxidative phosphorylation. MRCD is the most frequently congenital metabolic disease. The frequency is 1/5000 births. Half of MRCD cases are diagnosed in the neonatal period [1]. MRCDs in the neonatal period are fatal and show severe syndromes that are derived from multiple organs. Therefore, it is difficult to diagnose MRCD in the neonatal period. These pathogeneses are still unclear [2].

Hemophagocytic lymphohistiocytosis (HLH) is a hyperinflammatory disorder in an uncontrolled and ineffective immune response. HLH occurs either as a primary form or as a secondary form [3]. MRCDs as a cause of secondary form HLH are very rare.

We could diagnose MRCD by measuring the activity of the mitochondrial respiratory chain enzyme. We encountered a preterm infant who had MRCD that might have caused HLH. In this paper, we discuss the way to diagnose MRCD and the correlation between MRCD and HLH.

## 2. Case Report 

**Figure 1:**
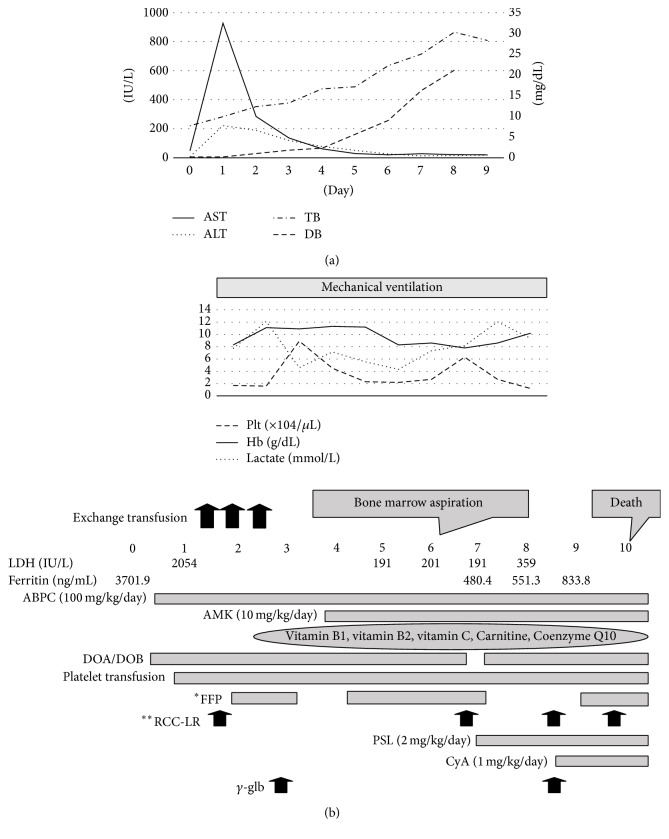
(a) Clinical course which is related to liver dysfunction. This figure shows the laboratory findings that are related to liver function. In a few days after birth, severe liver failure with a dominant increase in unconjugated bilirubin was evident. After that, aspartate aminotransferase and alanine aminotransferase levels were decreased; on the other hand, conjugated bilirubin level was increased. (b) Clinical course while she was hospitalized. This figure shows the clinical course of the patient. Thrombocytopenia did not improve after continuous platelet transfusion. Administration of vitamin B1, vitamin B2, vitamin C, Carnitine, and Coenzyme Q10 is a supporting therapy for mitochondrial disease. ^*∗*^FFP: fresh frozen plasma. ^*∗∗*^RCC-LR: red cell concentrates-leukocyte reduced.

A 39-year-old woman with no gestation and no parturition became pregnant by intracytoplasmic sperm injection. There was no family history of sudden death or consanguineous marriage. At 34 weeks and 4 days of gestation, fetal hydrops (pleural effusion, ascites, and subcutaneous edema) was recognized. The infant was delivered by emergency caesarean section due to a nonreassuring fetal status. The infant was intubated due to grunting and transferred to our NICU. Apgar scores were 7 (1 min)/9 (5 min). Her weight was 2264 g (appropriate for dates). Her muscle tone was low. Mild retractions with no murmur and no rale were recognized. Abdominal finding was the distention with hepatosplenomegaly. No abnormal findings existed. We show laboratory findings in Table 1. Lactic acidosis had continued, and jaundice appeared within 24 hours of birth. Disseminated intravascular coagulation (DIC) was recognized. The ammonia, C-reactive protein, and ferritin levels increased. On day 1, we measured cytokines. The findings were the following: IL-6 level 1398.3 pg/mL, IL-8 21675.0 pg/mL, INF-*γ* 5> pg/mL, and TNF-*α* 8.2 pg/mL. The findings showed hypercytokinemia. We performed an exchange transfusion three times (twice on day 1 and once on day 2) for the early onset jaundice. The patient had prolonged lactic acidosis, mild hyperammonemia, and liver failure that was similar to the presentation of Reye syndrome. Laboratory change, which is related to liver failure, is shown in Figure 1(a). Neonatal mass screening by tandem mass spectrometry, amino acid analysis, and urine organic acid analysis showed normal findings. From the above results, we suspected mitochondrial disease and began to administer vitamin B1, vitamin B2, vitamin C, Carnitine, and Coenzyme Q10 on day 2. On day 5, in the blood, lactate was 39.0 mg/dL, and pyruvic acid was 1.16 mg/dL (lactate/pyruvic acid ratio of 33.2 > 20). In the cerebral spinal fluid, lactate was 43.6 mg/dL, and pyruvic acid was 1.73 mg/dL (lactate/pyruvic acid ratio of 25.2 > 20). These results were also suggesting that she was mitochondrial disease. Thrombocytopenia and anemia did not improve. Specifically, thrombocytopenia was severe, and the patient received continuous platelet transfusion (Figure 1(b)). On day 6, we performed bone marrow aspiration, because mitochondrial disease was not enough for explaining pathogenesis of thrombocytopenia. We recognized a large amount of hemophagocytic macrophages, and we could not detect any evidence of malignancy. She was afebrile. However, the splenomegaly continued from admission. Other examination findings were as follows: WBC 11.4 × 10^3^/*μ*L, Hb 7.9 g/dL, Plt 2.4 × 10^4^/*μ*L, fibrinogen 74 mg/dL, and soluble IL-2 receptor 3805 U/mL. Although we could not measure fasting triglycerides and NK cell activity, our case was diagnosed as hemophagocytic lymphohistiocytosis (HLH), because she had five criteria of Revised Diagnostic Guidelines for HLH [4]. We began to administer prednisolone (2 mg/kg/day) on day 6 and cyclosporine (1 mg/kg/day) on day 8 to treat HLH. We also administered immunoglobulin (500 mg/kg) on day 8. On day 10, the patient died due to uncontrollable HLH, and an autopsy was performed after parental consent was given to perform the autopsy on the whole body except for her brain.

We took samples from the liver, muscle, and heart, and they were preserved at −80°C. Activities of the mitochondrial respiratory chain complexes I, II, III, and IV were assayed as described previously [5]. In these assays, citrate synthase (CS) was used as a housekeeping mitochondrial enzyme marker. The percent ratio of complex I activity to CS activity was decreased in all tissues (Table 2). We diagnosed MRCD (mitochondrial complex I deficiency) based on the diagnostic criteria given by Bernier et al. [6].

According to the results above, she was diagnosed as having MRCD with HLH. We could not detect an antecedent infection (Table 3). Although the mutation of* PRF1* is the most common genetic mutation of primary HLH in Japan, she had no mutation of* PRF1*.

## 3. Discussion

We determined two important clinical issues. First, it is useful to measure the activity of the mitochondrial respiratory chain enzyme for diagnosing MRCD. Second, MRCD, which has a severe clinical course, may be related to HLH.

The diagnostic criteria given by Bernier et al. is useful for diagnosing MRCD [6]. This diagnosis requires one of the following: enzymology, histology, functional assay, or molecular assay.

Enzymatic diagnosis of MRCD is achieved by the activity of the mitochondrial respiratory enzyme. We can accurately measure the activity of the mitochondrial respiratory chain enzyme, as we appropriately take a sample and immediately preserve it at −80°C [7]. We can determine the biological function of the mitochondria by measuring the activity of the mitochondrial respiratory chain enzyme. In histology, the fact that there are >2% ragged red fibers in the skeletal muscle is necessary for diagnosis; however, ragged red fibers are not common in children. Functional assays require the culture of fibroblasts, which takes a few months. Molecular assays are sometimes difficult. Calvo et al. reported exome sequencing of 42 MRCD patients, and only 24% of these patients had mutations in genes that have previously been linked to disease. 31% of them had mutations in nuclear genes that had never been linked to disease. 45% of them did not have any mutations [8]. Kohda et al. reported a comprehensive genetic analysis about 142 childhood-onset MRCDs. It revealed that 40 patients (28.2%) did not have any mutation in nuclear gene, mitochondrial DNA, and chromosome [9].

HLH might be secondary to MRCD. However, it is possible that HLH causes MRCD. It is well known that tumor necrosis factor (TNF) injures mitochondria and induces reactive oxygen species (ROS) production [10]. In our case, TNF-*α* was not so high. Therefore, it is difficult to interpret our case as secondary mitochondrial dysfunction due to hyperexcretion of TNF-*α*. In animal models, when the mitochondrial complex I is inhibited, the mitochondrial ROS production increases [11, 12]. Weinberg et al. reported that inflammatory cytokines are activated by mitochondrial generated ROS [13]. Therefore, in our case, we hypothesize that mitochondrial complex I deficiency causes ROS production, and HLH is induced by hypercytokinemia following the ROS.

Half of MRCD cases are diagnosed in the neonatal period, and 35% of the patients were diagnosed as having lethal infantile mitochondrial disorders [1]. As the symptoms of MRCD are nonspecific, the diagnosis is sometimes difficult. Therefore, MRCD may be the cause of the cryptogenic neonatal deaths that have severe clinical courses. HLH may be related to MRCD, which has a severe clinical course.

Cases involving both MRCD and HLH are rare, and we have experienced only one case. We must inform neonatologists about MRCD, because onset of MRCD is during the neonatal period. Then a further study of MRCD should be conducted.

## 4. Conclusion

We recommend that an assay of the mitochondrial respiratory chain enzyme should be performed if mitochondrial disease is suspected. MRCD may be related to the pathogenesis of secondary HLH.

## Figures and Tables

**Table 1:** Laboratory findings on admission.

CBC	WBC	25.3 × 10^3^/*μ*L
	Band	10%
	Seg	28.5%
	Lymph	47%
	Mono	11%
	Eosino	1.0%
	Baso	0%
	Blast	0%
	RBC	2.34 × 10^6^/*μ*L
	*Hb *	*8.3 g/dL*
	Ht	25.1%
	*Plt*	*1.7 × *10^4^ */μL*
	Ret	54.1‰

Coagulation	PT	37.1 sec
	**APTT**	**110.7 sec**
	*Fib*	*40 mg/dL*
	HPT	22.6%
	**FDP**	**25.1 ** ***μ*** **g/dL**
	*AT3*	*8.8%*

Biochemistry findings	**T-Bil**	**7.7 mg/dL**
	D-Bil	0.2 mg/dL
	AST	47 IU/L
	ALT	6 IU/L
	CK	69 IU/L
	BUN	7 mg/dL
	Cr	0.61 mg/dL
	Na	138 mmol/L
	K	4.2 mmol/L
	Cl	109 mmol/L
	Ca	9.3 mg/dL
	P	5.7 mg/dL
	TP	3.2 g/dL
	*ALB*	* 2.0 g/dL*
	**NH**_**3**_	**156 ** ***μ*** **g/dL**
	**Ferritin **	**3701.9 ng/mL**
	**CRP**	**1.23 mg/dL**

Blood type	O Rh(+)	

Immunological	*IgG*	*333 mg/dL *
	IgA	0 mg/dL
	IgM	12 mg/dL
	Direct Coombs test	(—)

Peripheral smear	Spherocytosis	(—)
	Elliptocytosis	(—)

Blood gas analysis (artery intubated)	pH	7.340
	O_2_	154.3 mmHg
	CO_2_	24.5 mmHg
	*HCO*_3_^−^	*12.9 mmol/L*
	*BE*	*−10.8 mmol/L*
	**Lac **	**70.1 mg**/**dL**
	**Anion gap**	**16.1**

Chromosomal test (G band)	46,XX	

This table shows all laboratory findings on admission.

Bold font: high; italic font: low.

**Table:** (a) Liver

%	Co I^*∗*^	Co II^*∗∗*^	Co III^*∗∗∗*^	Co IV^†^	CS^††^
Patient					
% of normal	15	31	78	100	108
CS ratio (%)	13	28	68	88	
Co II ratio (%)	46		242	252	

**Table:** (b) Muscle

%	Co I	Co II	Co II + III	Co III	Co IV	CS
Patient						
% of normal	17	54	57	51	35	63
CS ratio (%)	26	86	89	83	52	
Co II ratio (%)	30		100	94	57	

**Table:** (c) Heart

%	Co I	Co II	Co II + III	Co III	Co IV	CS
Patient						
% of normal	15	89	53	159	63	112
CS ratio (%)	14	79	46	142	57	
Co II ratio (%)	26		76	220	46	

This table shows the activity of the mitochondrial respiratory chain enzyme (I–IV) in the liver, muscle, and heart.

^*∗*^Co I: complex. ^*∗∗*^Co II: complex II. ^*∗∗∗*^Co III: complex III. ^†^Co IV: complex IV. ^††^CS: citrate synthase.

**Table 3:** Laboratory findings about antecedent infection.

Infection	Blood	
	Toxoplasma	IgM (−)
	Parvovirus B19	IgM (EIA) (−), PCR (−)
	Rubella	IgM (EIA) (−), PCR (−)
	Herpes simplex virus	IgM (EIA) (−), PCR (−)
	Cytomegalovirus	IgM (EIA) (−), PCR (−)
	Epstein-Barr virus	PCR (−)
	Urine	
	Cytomegalovirus	PCR (−)
	Primary isolation of virus (day 7)	(−)
	Feces · throat	
	Primary isolation of virus (day 7)	(−)

This table shows the laboratory findings regarding antecedent infection. We took samples for primary isolation of virus on day 7. The other samples were taken on admission. All findings were negative.
